# A dual-species co-cultivation system to study the interactions between *Roseobacters* and dinoflagellates

**DOI:** 10.3389/fmicb.2014.00311

**Published:** 2014-06-25

**Authors:** Hui Wang, Jürgen Tomasch, Michael Jarek, Irene Wagner-Döbler

**Affiliations:** Helmholtz-Centre for Infection ResearchBraunschweig, Germany

**Keywords:** symbiosis, dinoflagellates, *Roseobacter*, polyhydroxyalkanoates, DMSP, vitamin B 12, transcriptome

## Abstract

Some microalgae in nature live in symbiosis with microorganisms that can enhance or inhibit growth, thus influencing the dynamics of phytoplankton blooms. In spite of the great ecological importance of these interactions, very few defined laboratory systems are available to study them in detail. Here we present a co-cultivation system consisting of the toxic phototrophic dinoflagellate *Prorocentrum minimum* and the photoheterotrophic alphaproteobacterium *Dinoroseobacter shibae*. In a mineral medium lacking a carbon source, vitamins for the bacterium and the essential vitamin B_12_ for the dinoflagellate, growth dynamics reproducibly went from a mutualistic phase, where both algae and bacteria grow, to a pathogenic phase, where the algae are killed by the bacteria. The data show a “Jekyll and Hyde” lifestyle that had been proposed but not previously demonstrated. We used RNAseq and microarray analysis to determine which genes of *D. shibae* are transcribed and differentially expressed in a light dependent way at an early time-point of the co-culture when the bacterium grows very slowly. Enrichment of bacterial mRNA for transcriptome analysis was optimized, but none of the available methods proved capable of removing dinoflagellate ribosomal RNA completely. RNAseq showed that a phasin encoding gene (*phaP_1_*) which is part of the polyhydroxyalkanoate (PHA) metabolism operon represented approximately 10% of all transcripts. Five genes for aerobic anoxygenic photosynthesis were down-regulated in the light, indicating that the photosynthesis apparatus was functional. A betaine-choline-carnitine-transporter (BCCT) that may be used for dimethylsulfoniopropionate (DMSP) uptake was the highest up-regulated gene in the light. The data suggest that at this early mutualistic phase of the symbiosis, PHA degradation might be the main carbon and energy source of *D. shibae*, supplemented in the light by degradation of DMSP and aerobic anoxygenic photosynthesis.

## Introduction

Phytoplankton provide roughly 50% of the carbon fixed by photosynthesis to the global carbon cycle (Field et al., 1998). Eukaryotic phytoplankton often live in close association with bacterial symbionts that colonize the phycosphere, thrive on compounds excreted by the algae, and fulfill a variety of functions, including supply of essential vitamins or production of growth promoting compounds; they can also kill the algae by excreting algicidal compounds (Paul and Pohnert, 2011) or compete with it for micronutrients (Bratbak and Thingstad, 1985; Amin et al., 2012). To obtain a causal understanding of those interactions, experimental systems are required where cultivation media are defined and reproducible growth of the two species in co-culture can be obtained. Ideally, each species can also be cultivated in mono-species culture so that those two conditions can be compared. Setting up such systems is time-consuming and difficult, which may explain why there are so few available (Kumar et al., 2011; Angelis et al., 2012; Buhmann et al., 2012; Paul et al., 2013).

The *Roseobacters* are heterotrophic *Alphaproteobacteria* with high abundance in the marine ecosystem and large metabolic diversity (Buchan et al., 2005). They are very important contributors to the global carbon cycle because of their involvement in the assimilation of the dissolved organic matter (DOM) produced by phytoplankton and their ability to utilize aerobic anoxygenic phototrophy (AAnP) and carbon monoxide (CO) oxidation in the light to supplement their heterotrophic growth (Moran and Miller, 2007; Mou et al., 2008). Members of the *Roseobacter* clade also play a crucial role in the global sulfur cycle by transforming both inorganic and organic sulfur compounds. Most importantly, they are able to cleave the volatile dimethyl sulfide (DMS), which is a cloud condensation nucleus, from the algal osmolyte dimethylsulfonionpropionate (DMSP) (Howard et al., 2006; Moran et al., 2012). Many members of the *Roseobacter* clade were reported to be associated with marine algae (Gonzalez et al., 2000; Riemann et al., 2000; Alavi et al., 2001; Allgaier et al., 2003; Jasti et al., 2005), suggesting potential interactions between *Roseobacters* and marine algae. This is supported by a phylogenomic analysis, which shows that the *Roseobacter* lineage diverged from the SAR11 lineage approximately 260 million years ago, and that its first episode of diversification occurred approximately 196 million years ago, concurrent with the Mesozoic radiation of eukaryotic phytoplankton (dinoflagellates, coccolithophorids, and diatoms) (Luo et al., 2013).

The interaction between algae and *Roseobacters* can be mutualistic, antagonistic, or shift between both. For example, Seyedsayamdost et al. (2011) found that *Phaeobacter inhibens* BS107 in the presence of *p*-coumaric acid produces an algicidal compound termed Roseobacticide (Seyedsayamdost et al., 2011). The *p*-coumaric acid is a degradation product of lignin and is released by ageing diatoms. *P. inhibens* also produces tropodithietic acid (TDA), an antibacterial compound (Geng and Belas, 2011). Thus, the relationship between the coccolithophore *Emiliana huxleyi* and *P. inhibens* might shift from a mutualistic stage, where the bacteria protect the algae from other bacteria through the synthesis of TDA, to a pathogenic stage where algal lysis is induced by the Roseobacticides (Seyedsayamdost et al., 2011). Algicidal compounds are also produced by other members of the *Roseobacter* clade (Riclea et al., 2012).

The *Roseobacter* strain *Ruegeria* sp. strain TM1040 (originally termed *Silicibacter* sp. TM1040) was isolated from a culture of the heterotrophic dinoflagellate *Pfiesteria piscicida* (Alavi et al., 2001). It has been shown to catabolize DMSP produced by *Pfiesteria* (Miller and Belas, 2004). Many *Roseobacters* harbor pathways for DMSP degradation and through the emitted metabolite DMS indirectly influence the abundance of cloud condensation nuclei in the atmosphere (Dickschat et al., 2010). TM1040 additionally is positively chemotactic toward DMSP (Miller and Belas, 2006). *Pfisteria* requires the presence of TM1040 for growth, indicating an obligate symbiotic relationship between these two organisms. Thus, the symbiosis between TM1040 and *P. piscicida* has been used as a model to study the interactions between *Roseobacter* and phytoplankton. Recently it was shown that a mutation in the flagella gene *flaC* resulted in population heterogeneity with respect to flagellation (Sule and Belas, 2013). Upon stimulation with *p*-coumaric acid, a compound termed Roseobacter Motility Inducer (RMI) was excreted by TM1040 which restored motility and flagella synthesis in the TM1040 mutants but had a detrimental effect on algal motility and growth (Sule and Belas, 2013). Interestingly, TM1040 is one of the few *Roseobacter* strains that lacks the luxI/luxR type genetic elements for cell-cell communication through acylated homoserine lactones (AHLs) (Cude and Buchan, 2013).

*D. shibae* DFL12, another model organism of the *Roseobacter* clade, was isolated from the phototrophic marine toxic dinoflagellate *Prorocentrum lima* (Biebl et al., 2005). This bacterium possesses the genetic machinery for *de novo* vitamin B_12_ biosynthesis (Wagner-Döbler et al., 2010). Traits that might also be important for symbiosis with the algae are flagella synthesis and expression of the type IV secretion system, both of which are under the control of AHLs (Patzelt et al., 2013). *D. shibae* is photoheterotrophic, and diurnal changes in the light regimen had a profound influence on the expression of the photosynthesis gene cluster, genes related to central metabolism, and oxidative stress response related genes (Tomasch et al., 2011). *D. shibae* strains were also isolated from another dinoflagellate, *Alexandrium ostenfeldtii* and several other algal species, and therefore it is likely that its interaction with algae is not species-specific (Wagner-Döbler et al., 2010). Therefore, in previous work from our laboratory, we used an axenic culture of *Prorocentrum minimum*, closely related to *P. lima*, to study the interactions between *D. shibae* and dinoflagellates. *P. minimum* is a toxic red tide-forming dinoflagellate with a global distribution (Heil et al., 2005) and it is vitamin B_12_ auxotrophic. We have demonstrated a mutualistic association between *D. shibae* and *P. minimum*, in which *D. shibae* provides *P. minimum* with essential vitamins (B_1_ and B_12_) and *P. minimum* in return provides *D. shibae* with carbon sources and vitamins (B_3_ and 4-aminobenzoic acid) essential for growth of the bacterium (Wagner-Döbler et al., 2010).

Some known and putative molecular mechanisms involved in symbiosis between *Roseobacters* and phytoplankton have recently been reviewed (Geng and Belas, 2010). However, little is known about the role of light for this symbiosis. Interestingly, it was reported that glycolate, a byproduct of photorespiration by photoautotrophs, can be assimilated by bacteria harboring the glycolate oxidase gene *glcD* (Lau and Armbrust, 2006). Moreover, field studies during a phytoplankton spring bloom showed variation in the abundance of *glcD* mRNA transcripts over the diel cycle, with a consistent increase in transcripts during the day when glycolate production reached its maximum (Lau et al., 2007; Amin et al., 2012). In a recent study, Paul et al. (2013) showed that the metabolism of a diatom (*Thalassiosira pseudonana*) was strongly modified in contact-free co-culture with *D. shibae*.

In this study, we established a highly reproducible laboratory model system for studying the interaction of *P. minimum* and *D. shibae* and focus on gene expression of the bacterium. We show the population dynamics of *P. minimum* and *D. shibae* in co-culture by enumerating both organisms using flow cytometry. The co-culture reproducibly went from mutualism to pathogenesis: The mutualistic phase, where both bacteria and algae profit, was followed by a pathogenic phase where the bacteria induce death of the algae. In the present study we focus exclusively on the early mutualistic phase of the co-culture where *D. shibae* does not actively grow and ask which genes are transcribed in these cells and what effect the light-dark cycle has on the transcriptome. The aim was to establish methods for transcriptome analysis under the most challenging conditions, i.e., when cell numbers are low and the cells are dividing very slowly. We used both RNAseq and microarray analysis to study these questions.

## Materials and methods

### Algal culture

The axenic culture of *Prorocentrum minimum* strain CCMP 1329 used in this work was obtained from the Provasoli-Guillard National Center for Marine Algae and Microbiota (NCMA, formerly the Provasoli-Guillard National Center for Culture of Marine Phytoplankton, CCMP). *P. minimum* CCMP 1329 was cultivated in L_1_ medium prepared according to Guillard and Hargraves (1993) with the modifications that synthetic ocean water (Sunda et al., 2005) was used instead of natural seawater, and Na2SiO3 · 9H2O was omitted, since *P. minimum* does not require silica. All cultures were grown in 100 ml batches in 300 ml Erlenmeyer flasks at 22°C under a 12:12 h light-dark cycle with a light intensity of about 40 μmol photons m^−2^s^−1^. The *P. minimum* culture was maintained in our lab by transferring 1% of the culture volume to fresh medium every 4 weeks. Lack of contaminating bacteria was checked by streaking aliquots on LB and Difco marine agar 2216 (MB) plates.

### Bacterial culture

*D. shibae* DFL-12 was grown at 30°C and 160 rpm in a chemically defined sea water medium (SWM) supplemented with 5 mM succinate, prepared as described previously (Tomasch et al., 2011).

### Algae-bacteria co-culture

The pre-culture of *D. shibae* for co-cultivation experiments was grown in SWM to the late exponential phase, washed once by centrifugation at 5000 rpm for 5 min, and resuspended in L_1_ medium lacking vitamin B_12_ (L_1_-B_12_). The co-culture was obtained by adding bacterial cells up to a final density of 10^7^ cells/ml to the culture of *P. minimum* immediately after subculturing it in fresh L_1_-B_12_ medium with an initial density of approximately 2000 cells/ml. The cell numbers of both bacterial and algal pre-cultures were determined by flow cytometry (see below). In addition to this experimental set-up, three control cultures were prepared: (1) *P. minimum* alone in L_1_-Si medium with B_12_; (2) *P. minimum* alone in L_1_-Si medium without B_12_; (3) *D. shibae* alone in L_1_-Si medium without B_12_. The co-culture and all control cultures were prepared in triplicate and incubated under the same conditions as the algal culture.

### Cell counting

Growth of algae and bacteria under all conditions was followed by cell counting using a BD FACS Canto flow cytometer (BD Biosciences, San Jose, CA, USA). *P. minimum* was identified according to its chlorophyll autofluorescence and *D. shibae* was identified by staining with SYBR Green I (Molecular Probes, Leiden, The Netherlands). Both chlorophyll and SYBR Green I are excited with the 488 nm excitation laser line and emit at 695 nm (far red) and 519 nm (green), respectively. Depending on the cell size and fluorescence of these two organisms, typical settings of the flow cytometer for *P. minimum* were as follows: forward scatter (FSC) = 300, side scatter (SSC) = 250, far red fluorescence (PerCP-Cy5.5) = 300. For *D. shibae* stained with SYBR Green I the typical settings were as follows: FSC = 700, SSC = 400, green fluorescence (FITC) = 400.

A 1 ml sample from each control and experimental condition was taken in the light period and fixed with 25% glutaraldehyde to a final concentration of 2% for about 15 minutes at room temperature. For determining the population density of *P. minimum* in the co-culture a sample of 500 μl was analyzed, while for *D. shibae* the sample was diluted to an appropriate density (<1000 events s^−1^) with PBS (pH 7.0) to avoid coincidence and then prior to analysis the SYBR Green I was added at a final concentration of 10^−4^ of the stock reagent. Each sample was analyzed for approximately 2 min at a flow rate of 1.2 μl/s determined according to Marie et al. (2005). After analysis the acquisition time and the number of cells acquired were recorded to calculate the population density for each sample (Marie et al., 2005).

### Sampling

All cultures were monitored for 36 days. Sampling for cell counting by flow cytometry was performed every 3 days for the first 24 days and then every 6 days till the end of the experiment. For transcriptome analysis of the co-culture in the light and in the dark, sampling was performed at day 12, when the algae had reached the late exponential growth phase. At 5 h after the onset of the light or the dark period, respectively, 50 ml of the cultures were pelleted at 5000 rpm for 10 min and transferred into 2 ml Eppendorf tubes. The cell pellets were then covered with 1 ml Trizol reagent (Ambion, Life Technologies, Carlsbad, CA, USA), snap frozen in liquid nitrogen and stored at −70°C until RNA isolation.

### RNA isolation

For RNA extraction, the sample was thawed at room temperature and then transferred to a cryotube filled with 0.3 g acid washed glass beads. The cells were homogenized using the FastPrep-24 instrument (MP Biomedicals, California, USA) at 6.0 m/s for 3 min and then incubated for 5 min at room temperature. Samples were centrifuged at 12,000 g for 10 min at 4°C and the supernatants were transferred to fresh tubes, followed by the addition of 100 μl of 1-bromo-3-chloropropane (BCP, Sigma, Germany) and incubation for 10 min at room temperature. Samples were centrifuged at 12,000 g for 10 min at 4°C, after which the aqueous phase was transferred to new tubes and mixed with 500 μl of absolute ethanol. Extracts were applied to RNeasy spin columns (RNeasy mini kit, Qiagen, Hilden, Germany) and processed according to the manufacturer's instructions. In addition, samples were treated with DNAse I (Qiagen, Hilden, Germany). Removal of genomic DNA was verified via PCR using total RNA as template. The concentration of the RNA was quantified using a NanoDrop spectrophotometer (Peqlab, Erlangen, Germany) and the RNA integrity was assessed using a Bioanalyzer 2100 (Agilent, Santa Clara, USA).

### Ribosomal RNA depletion

Two different approaches have been tested. The data presented in this manuscript has been generated from samples using the following strategy: The Oligotex mRNA kit (Qiagen, Hilden, Germany) was used to isolate the algal mRNA. For isolation of *D. shibae* mRNA, the MICROBEnrich kit (Ambion, Life Technologies, Darmstadt, Germany) was used to remove the eukaryotic rRNA, and the MICROBExpress kit (Ambion, Life Technologies, Darmstadt, Germany) was used to remove the prokaryotic rRNA, according to the manufacturer's instructions. A second approach resulted in an mRNA proportion of about 0.01%. These samples have therefore not been analyzed further: PolyATract System IV (Promega, Madsion, WI, USA) was used for isolation of algal mRNA according to the manufacturer's instructions. For isolation of the bacterial mRNA Ribo-Zero Magnetic Kit for both plant leaf and gram-negative bacteria (Epicenter, Madsion, WI, USA) were used according to the manufacturer's instructions. The final purification of the eluted mRNA was performed using ethanol precipitation.

### RNA sequencing and data analysis

Libraries of 300 bp for RNA sequencing were prepared using the mRNA-Seq sample preparation kit (Illumina, San Diego, CA, USA) according to the manufacturer's instructions. Quality control of the prepared libraries was validated using the Agilent Bioanalyzer (Agilent Technologies, Santa Clara, USA). Cluster generation was performed using the Illumina cluster station. Sequencing was done on the Genome Analyzer IIx (Illumina, San Diego, CA, USA) following a standard protocol. The fluorescent images were processed to sequences and transformed to FastQ format using the Genome Analyzer Pipeline Analysis software 1.8 (Illumina, San Diego, CA, USA). The sequencing output (36 bp single end short reads) of the Genome Analyzer IIx was controlled for general quality features using the fastq-mcf tool of ea-utils (http://code.google.com/p/ea-utils) and was mapped against the genome sequence of *D. shibae* (NC_009952.1, NC_009955.1 NC_009956.1, NC_009957.1, NC_009958.1, and NC_009959.1) using Burrows-Wheeler Aligner (BWA) v 0.5.9-r16 (Li and Durbin, 2009). Statistical analysis of the mapped read counts was performed in the R environment using basic functions (http://CRAN.R-project.org). Sampling of a read counts dataset was performed using the rrarefy-function of the vegan package (http://CRAN.R-project.org/package=vegan).

### Microarray experiment and data analysis

To analyze the differences in gene expression of *D. shibae* associated with *P. minimum* in the light and dark, a microarray analysis was performed according to the method described by Tomasch et al. (2011). In brief, samples from the light were labeled with Cy3 and samples from the dark were labeled with Cy5. 2 μg RNA from each sample was labeled using the Universal Linkage System (ULS) (Kreatech, Amsterdam, The Netherlands) according to the manufacturer's manual. Five hundred ng of each labeled RNA sample were fragmented and hybridized to the microarray according to Agilent's two-color microarray protocol. Median foreground and background spot intensities of the Cy3 and Cy5 channel were loaded into the R environment and processed using the LIMMA package (Smyth, 2005). Background signals were subtracted using the normexp method (Ritchie et al., 2007). Cy3 and Cy5 signals were Loess normalized and finally quantile normalization was performed on both microarrays. Signals from replicate probes for single genes were averaged. A linear model was fitted for each comparison of interest. The obtained *p*-values were adjusted for false discovery rate (fdr) using the method by Benjamini and Hochberg (1995).

### Data access

Raw and processed RNAseq and microarray data have been deposited in the gene expression omnibus database (http://www.ncbi.nlm.nih.gov/geo/) under the accession number GSE53967.

## Results and discussion

### Population dynamics of *D. shibae* and *P. minimum* in co-culture

**Figure 1:**
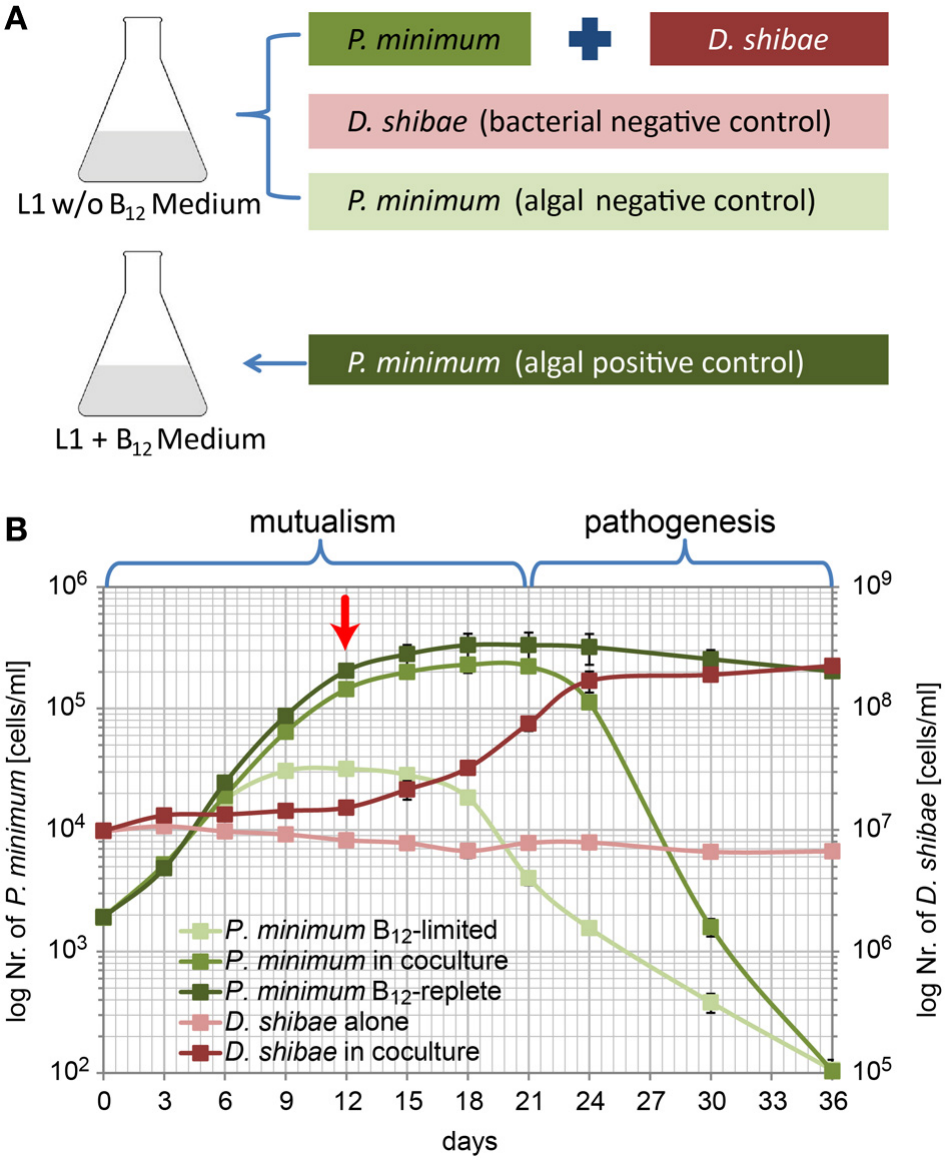
**Population dynamics of *P. minimum* and *D. shibae* in co-culture. (A)** Experimental setup of the co-cultivation experiment. **(B)** Growth of *P. minimum* and *D. shibae* determined by flow cytometric analysis. In the mutualistic phase (day 0–day 21) both organisms profit from the co-cultivation in a medium where single organisms are not able to grow. In the pathogenic phase (day 21–day 36) the algae are killed by the bacteria, which continue to grow. The red arrow indicates the sampling time point. Data represent mean and standard deviation of three biological replicates.

Our laboratory model system for investigating the interactions between *D. shibae* and *P. minimum* was established based on the evidence that *P. minimum* relies on vitamin B_12_ produced by *D. shibae* and provides carbon sources and essential vitamins for the growth of its mutualistic partner (Wagner-Döbler et al., 2010). It is not known which carbon sources are provided by the dinoflagellate. *D. shibae* requires three essential vitamins, biotin, niacin, and 4-amino-benzoic acid (Biebl et al., 2005). Biotin is also needed by the dinoflagellate and therefore is present in the cultivation medium. Niacin and 4-amino-benzoic acid are not present in the cultivation medium. Thus, we must assume that the dinoflagellate supplies them to the bacterium. Therefore, for the co-cultivation experiment *D. shibae* and *P. minimum* were grown in a medium lacking B_12_. Moreover, this medium also did not contain organic carbon sources and essential vitamins for *D. shibae* (niacin, 4-aminobenzoic acid). *D. shibae* alone and *P. minimum* alone were cultivated in the same L_1_-B_12_ medium as bacterial negative and algal negative controls, respectively. Additionally, *P. minimum* alone in the medium supplemented with synthetic vitamin B_12_ (L_1_ + B_12_) was cultivated as an algal positive control (Figure 1A).

In L_1_-B_12_ medium neither a carbon source and essential vitamins for the bacteria nor the vitamin B_12_ for the algae was present. *D. shibae* alone was not able to grow in this medium as shown in Figure 1B. *P. minimum* alone showed some growth at the beginning of the experimental period due to medium takeover from the algal pre-culture still containing traces of vitamin B_12_. However, the algae did not reach a high cell density, entered into stationary phase already at day 9 and its density fell to approximately 100 cells/ml at the end.

In the co-culture, the number of *D. shibae* cells showed a slight initial increase (day 0 to 12) followed by a remarkable increase from day 12 to day 24. During this period the growth of the algae slowed down. From day 24 onwards the number of algal cells decreased. *D. shibae* reached a cell density of 1.7 × 10^8^ cells/ml by day 24 which remained quite stable until the end of the experiment. These observations suggest that carbon sources and the essential vitamins that *D. shibae* required for growth were mainly provided in the stationary phase of *P. minimum* growth.

*P. minimum* in the co-culture, obviously dependent on the vitamin B_12_ produced by *D. shibae*, grew in a similar way as the algal positive control with a period of high growth rate (day 0–12) and a period with decreased growth until the stationary phase was reached at about day 18 with 2.3 × 10^5^ cells/ml. While the algal positive control remained at a stable density in the stationary phase until the end of the experiment, surprisingly, the number of *P. minimum* cells in the co-culture declined markedly from 2.2 × 10^5^ cells/ml by day 21 to as low as 1.0 × 10^2^ cells/ml at day 36. These results suggest that in the co-culture *D. shibae* has initially a stimulating effect on the growth of *P. minimum* (from day 0–18) but seems to induce cell death after the algae have reached the stationary phase (from day 21 onwards). Thus, a mutualistic phase (day 1–21) is followed by a pathogenic phase (day 21–36).

Our results suggest that *D. shibae*, while providing enough vitamin B_12_ to allow exponential growth of *P. minimum*, does not get enough C-compounds or limiting vitamins to actively grow at the beginning of the co-culture, but still enough to maintain a stable cell density. Only when the growth of *P. minimum* slowed down *D. shibae* actively started to grow. The amount of carbon and vitamins excreted by the algae during exponential growth might have been too low for the bacteria.

The decline of *P. minimum* cells in the second phase of the co-culture suggests that *D. shibae* might induce algal death. Thus, the interaction of *P. minimum* and *D. shibae* resembles the “Jekyll and Hyde” interaction that has recently been proposed but not yet been shown in co-culture for *E. huxleyi* and *P. inhibens* (Seyedsayamdost et al., 2011). However, algicidal compounds like those identified in *P. inhibens* and other *Roseobacter* strains (Riclea et al., 2012; Sule and Belas, 2013), remain to be identified in *D. shibae*.

### Testing of strategies for the enrichment of bacterial mRNA

**Figure 2:**
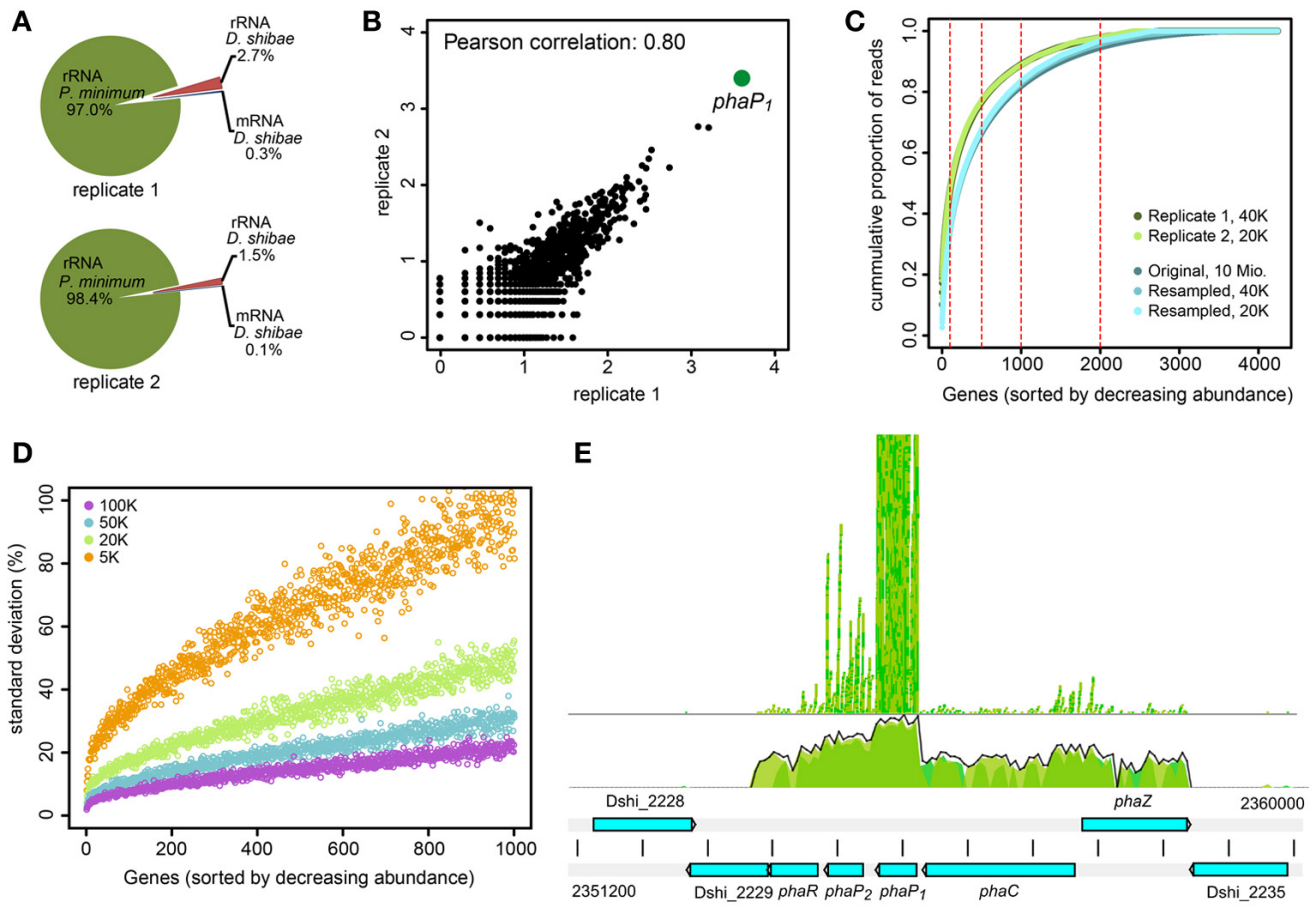
**RNA-sequencing of *D. shibae* in the light after 12 days of co-culture with *P. minimum*. (A)** Assigned reads from enriched RNA of two biological replicates to rRNA of *P. minimum*, rRNA and mRNA of *D. shibae*. **(B)** Scatterplot and Pearson correlation of read coverage for all genes with at least one mapped read in each biological replicate. Data are shown as log_10_ mapped reads. **(C)** Cumulative proportion of mapped reads per gene for replicate 1 (dark green) and replicate 2 (light green) of this study. The cumulative proportion of a dataset with high sequencing depth (approximately 10 Mio. reads) is shown for comparison (dark blue). Reducing the sequencing depth to 40,000 (blue) and 20,000 (light blue) reads does not change the read distribution. **(D)** Standard deviation of mapped reads proportion from the original value, when a library of approximately 10 million mapped reads is subsampled to match the library size of 100,000 to 5000 reads. The data represent deviations from 100 random sampling steps. Only the 1000 genes with the highest abundance are shown. **(E)** Read coverage of the *pha*-locus. *PhaC* to *phaR* are transcribed as an operon. Transcript abundance is highest for *phaP_2_* and in particular *phaP_1_*. *phaR*: Transcriptional regulator; *phaP_1/2_*: Phasin; *phaC*: PHA synthetase; *phaZ*: PHA depolymerase.

We selected two biological replicates of samples taken at day 12 in the light for RNAseq analysis. We first asked whether our approach to enrich bacterial mRNA was successful. We found that only a small fraction of the generated reads could be mapped to the genome of *D. shibae*. The data are summarized in Table 1 and Figure 2A. Only 40,488 (0.3%) and 20,171 (0.1%) of the reads for replicate one and two, respectively, mapped on protein coding sequences (Figure 2A). Thus, we had not been successful to substantially enrich bacterial mRNA. This may be due to the kits that we have used to deplete both the eukaryotic and prokaryotic rRNA in the co-culture. The MICROBEnrich kit used to remove the eukaryotic RNA is based on the 18S and 28S rRNA specific capture oligonucleotides designed for removing human, rat and mouse rRNA from host-bacteria RNA mixtures. Our results show that this approach was not effective in removing the *P. minimum* rRNA from the co-culture, most likely because dinoflagellate ribosomal RNA was not captured by those probes. Moreover, the subsequent further enrichment of *D. shibae* mRNA using the MICROBExpress kit was also not effective, with 90% and 93.8% rRNA reads remaining in replicate 1 and replicate 2, respectively. A recent study by Giannoukos et al. (2012) comparing 5 different rRNA removal methods showed a low efficiency of the MICROBExpress kit and a very high efficiency of the Ribo-Zero kit from Epicenter in 3 tested bacteria, among which *Rhodobacter sphaeroides* is very closely related to *D. shibae*. Thus, we tested the Ribo-Zero magnetic kit for gram-negative bacteria on a pure culture of *D. shibae* culture. The results showed that 50-70% rRNA could be removed applying this method (data unpublished). To remove the algal rRNA from the co-culture the Ribo-Zero magnetic kit for plant leaves was tested. Although the homology of the rRNA specific capture probe from Ribo-Zero plant to that of the *P. minimum* ribosomal RNA was 85% for the 18S rRNA and 83% for the 25S rRNA (data provided by the technical support of Epicenter), the efficiency of rRNA removal of *P. minimum* in the co-culture was still not improved (data unpublished). The number of mRNA reads was about 0.01% in these samples, thus it was even lower than that in the samples using the previous enrichment. However, using this strategy, we found that the sequencing depth improved with ongoing cultivation and increasing number of bacteria in the co-culture and reached more than 1 million mapped reads for the samples at day 24 (data unpublished). At this early stage of co-cultivation, the number of bacteria might be simply too low. Thus, other strategies have to be developed to enrich bacterial mRNA, e.g., physical separation of the two organisms through filtration. This method however would have the drawback that bacterial cells attached to algae would not be captured and that the mRNA is not stabilized during the filtration process. Another possibility would be cultivation in compartments separated through a membrane. This would allow studying interactions that do not require direct contact of both organisms (Paul et al., 2013).

**Table 1:** **Mapping statistics of RNA-seq data**.

**Fragment**	**Replicate 1**		**Replicate 2**	
	**No. of reads**	**% reads**	**No. of reads**	**% reads**
Counted fragments	420,586	3.0	259,621	1.6
Uniquely	40,488	0.3	20,171	0.1
Non-specifically	380,098	2.7	239,450	1.5
Uncounted fragments	13,590,745	97.0	16,239,551	98.4
Total fragments	14,011,331	100.0	16,499,172	100.0

### Transcriptome of *D. shibae* in the light as revealed by RNA sequencing

Although the obtained mRNA was only the “tip of the iceberg,” it might provide consistent and interesting observations. Both replicate samples showed a good Pearson correlation of 0.80 when all genes with at least one read in both samples were considered (Figure 2B). Strikingly, 10.1% (4108 reads) and 12.4% (2511 reads) of the reads mapped to one single gene, *phaP_1_*. Only few other genes showed a high expression level (Table 2). Around half of the reads mapped to only 100 genes and around 90% to 1000 genes (Figure 2C). As this distribution might be an artifact resulting from the low sequencing depth we sampled 40,000 and 20,000 reads from a dataset with a sequencing depth of 10 million reads (Bill et al., unpublished) and compared the distribution of the original and the reduced data. These data were obtained from a growing single-culture of *D. shibae*. The reduction of the sequencing depth indeed resulted in a bias of mapped reads toward higher expressed genes, but did not lead to dramatic changes of the read distribution (Figure 2C). Thus, it can be assumed that the observed is close to the actual distribution of expressed genes and does not represent an artifact.

**Table 2:** **Most highly expressed genes of *D. shibae* in the light after 12 days of co-cultivation with *P. minimum* determined by RNAseq**.

**Locus tag**	**Description**	**Replicate 1**		**Replicate 2**	
		**No. reads**	**% reads**	**No. reads**	**% reads**
Dshi_2232	Phasin, phaP1	4108	10.1	2511	12.4
Dshi_0043	Porin, Gram-negative type	1646	4.1	566	2.8
Dshi_1205	Hypothetical protein	1231	3.0	583	2.9
Dshi_1095	Aldehyde dehydrogenase	559	1.4	169	0.8
Dshi_0247	rpsU-divergently transcribed protein	341	0.8	288	1.4
Dshi_0661	Cytochrome c oxidase, cbb3-type	316	0.8	221	1.1
Dshi_2231	Phasin, phaP2	294	0.7	166	0.8
Dshi_2283	Phenylacetone monooxygenase	293	0.7	48	0.2
Dshi_0870	Transcriptional regulator, SARP family	291	0.7	74	0.4
Dshi_2181	Acyl carrier protein	282	0.7	91	0.5
Dshi_1968	Pyruvate dehydrogenase E1 component	263	0.6	180	0.9
Dshi_3371	Ribosomal protein S20	172	0.4	126	0.6
Dshi_3556	Sodium/solute symporter family protein	242	0.6	111	0.6

Since the selection of fragments for sequencing is a random process, we considered the error which might result from the very low sequencing depth of our samples. We resampled the 10 million mapped reads of the dataset by Bill et al. (unpublished) according to library sizes from 100,000 to 5000 reads and calculated the deviation of the proportion of the genes in the subsampled dataset from that of the original dataset. For each library size 100 re-samplings were performed and the standard deviation was calculated. The standard deviation increased with decreasing read coverage and this increase was extremely high for genes with a low coverage of reads (Figure 2D). However, even sampling of only 5000 reads from the original dataset did not lead to a high error for the most abundant genes. Thus, the high abundance of reads mapping to the phasin *phaP_1_* is probably not an artifact of the sequencing as a consequence of the small library size but represents the real abundance of this mRNA in the cell. The error introduced through low coverage of the genome would of course corrupt detection of weakly differentially expressed genes. Therefore, we decided not to sequence the corresponding samples taken in the dark.

The extremely high expression of *phaP_1_* (Dshi_2232) was remarkable, a second phasin-encoding gene, *phaP_2_*, was also highly expressed, so we asked for the role of the gene products. Phasins are small, amphiphilic proteins and are structural components of polyhydroxyalkanoate (PHA) granule membranes (Potter and Steinbüchel, 2005). PHAs are intracellular carbon and energy storage compounds produced by many prokaryotes (Anderson and Dawes, 1990). Phasins can contribute up to about 5% of total cellular protein (Wieczorek et al., 1995) and are considered to be involved in the regulation of PHA synthesis and degradation (York et al., 2001; Handrick et al., 2004; Kuchta et al., 2007). In the genome of *D. shibae* the genes involved in PHA biosynthesis and metabolism are clustered at two loci (Table 3). One locus contains genes coding for a PHA synthetase (*phaC*), two phasins (*phaP_1/2_*), a transcriptional regulator (*phaR*) and a PHA depolymerase (*phaZ)*. The first four genes are transcribed as an operon (Figure 2E). *PhaZ* is located next to *phaC*, on the opposite strand. Thus, both genes are transcribed from a bidirectional promoter region. The other locus contains genes encoding β-ketothiolase (*phaA*) and acetoactyl-CoA reductase (*phaB*). Both *pha* loci were actively transcribed in our experiment, although the expression levels of *phaP_1_* and *phaP_2_* were around 100 and 10 times higher than those of the other genes, respectively (Supplementary Material).

**Table 3:** **Similarity of *D. shibae* genes involved in PHA biosynthesis and metabolism to reference organisms**.

**Locus tag *D. shibae***	**Gene symbol**	**Description**	***Ralstonia eutropha* H16**			***Rhodobacter sphaeroides***		
			**GenBank**	***E*-value**	**Identity (%)**	**GenBank**	***E*-value**	**Identity (%)**
Dshi_2230	*phaR*	Transcriptional regulator	CAJ92575.1	1.0E−19	38	DQ003490.1	3.0E−83	68
Dshi_2231	*phaP_2_*	Phasin	–	–	–	DQ003489.1	2.0E−28	43
Dshi_2232	*phaP_1_*	Phasin	–	–	–	DQ003489.1	5.0E−42	70
Dshi_2233	*phaC*	PHB synthase	CAJ92572.1	3.0E−120	39	DQ003488.1	0.0E+00	67
Dshi_2234	*phaZ*	PHB depolymerase	CAJ92291.1	1.0E−79	38	DQ003491.1	0.0E+00	74
Dshi_3066	*phaA*	Beta-ketothiolase	CAJ92573.1	8.0E−140	60	DQ003486.1	0.0E+00	80
Dshi_3067	*phaB*	Acetylactyl-CoA reductase	CAJ93268.1	1.0E−66	49	DQ003487.1	1.0E−137	78

*– indicates no similarity found in reference organisms*.

As described above, the errors introduced by the extremely low coverage of our RNAseq runs influence the reliability of a comparative analysis except for the most highly expressed genes. Therefore, we did not sequence samples taken in the dark but used instead two-color microarray technology to compare the transcriptomes of *D. shibae* in the light and in the dark.

### Comparative analysis of the transcriptomes of *D. shibae* in the light and in the dark as revealed by microarray analysis

In our lab we use custom-made Agilent microarrays combined with direct labeling of total RNA, thus samples with a low amount of mRNA are routinely analyzed (Tomasch et al., 2011). We wondered if an even further decrease of mRNA in the sample below the normal amount might influence the analysis of differential gene expression.

**Figure 3:**
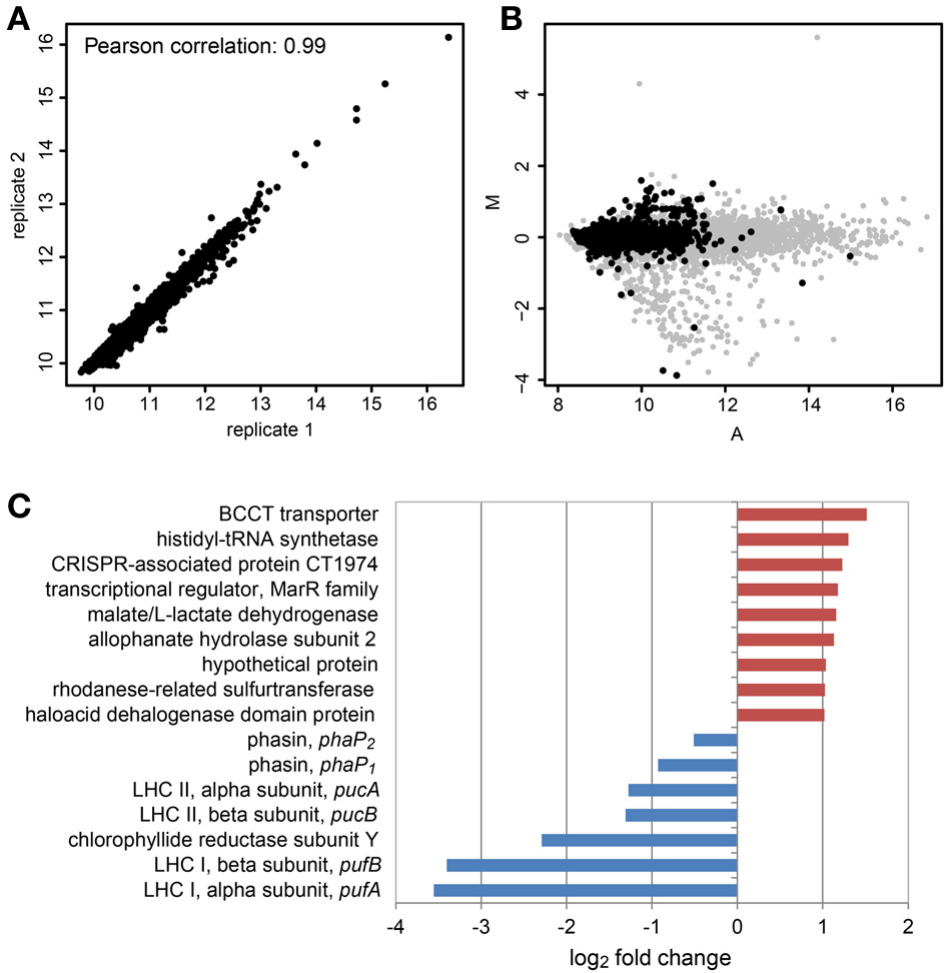
**Differential gene expression of *D. shibae* in the light compared to the dark after 12 days of co-culture with *P. minimum* using microarray analysis. (A)** Scatterplot and Pearson correlation of the mean log_2_ intensities of two microarrays with biological replicates from light and dark samples. **(B)** MA-plot obtained from microarray data of *D. shibae* in co-culture (black spots) compared with that of *D. shibae* in the exponential growth phase using nutrient-replete medium (gray spots). The distribution of intensities does not cover the whole dynamic range and only a small set of genes shows differential expression for the actual data. The *A*-values have been shifted to the left, to allow comparison of both datasets. M, log_2_ fold change; A, log_2_ mean intensity of both channels. **(C)** Differentially expressed genes in samples from the light compared to the dark. Red indicates up-regulation, blue indicates down-regulation in the light.

We compared one sample in the light and one sample in the dark on one microarray slide with a biological replicate of each sample on a second microarray slide. The mean signal intensities of both microarrays showed a Pearson correlation of 0.99, so reproducibility was high (Figure 3A). The signal intensity range however did not cover the whole dynamic range of the scanner, which can best be seen when the MA-plot of a sample is compared to that of a sample from an experiment with single culture samples (Figure 3B). This might be a result of the low amount of mRNA in the sample. This plot also demonstrates that only few genes had high signal intensities and that only few genes were differentially expressed.

When the cut-offs were set to an adjusted *p*-value lower than 0.01 and an absolute log_2_ fold-change higher than 1, only nine genes showed an increased and five genes showed a decreased expression in the light (Figure 3C), the latter ones being exclusively genes of the photosynthesis gene cluster (PGC). We had previously shown that this cluster is strongly down-regulated in the light when *D. shibae* is cultivated in a C-limited chemostat (Tomasch et al., 2011). The five genes of the PGC showing a significant repression in the light in the co-culture encoding structural proteins of both light-harvesting complexes and the Y subunit of the chlorophyllide reductase. These genes had also shown the highest change in gene expression in our previous experiment. Thus, these results are consistent with previously published data. Therefore, we conclude that the differential expression results obtained here are trustworthy. Both phasin genes were down regulated in the light, too, but to an extent that was below the cut-off. As the *p*-values were 0.11 for *phaP_1_* and 0.13 for *phaP_2_* these results should be interpreted with extreme caution. However, it has been shown in another *Dinoroseobacter* strain that with an excess of the carbon source, light has a positive effect on the formation of PHA in the cell (Xiao and Jiao, 2011). In our case when carbon is scarce, light might reduce the need to use internal PHAs as an energy source. Thus, the turnover rate of the PHA bodies and therefore the expression of the *phaP* genes might be reduced in the light.

The gene (Dshi_0579) with the highest activation in the light encoded a betaine-choline-carnitine-transporter (BCCT)-type protein. These transporters are ubiquitous in bacteria and often have a role in protection from osmotic stress by regulating the flux of organic osmolytes (Ziegler et al., 2010). In the marine bacterium *Halomonas* sp. HTNK1, it has been shown, that the BCCT-protein *dddT* mediates the uptake of DMSP that is further catabolized to 3-hydroxy-propionate and acetyl-CoA, thus providing a carbon and energy source (Todd et al., 2010). The *D. shibae* BCCT-type protein shares 33% identical and 50% similar amino acid residues with that of *Halomonas*, thus it is possible that it is also involved in the uptake of DMSP. A second gene that encodes a BCCT-protein (Dshi_2119) with a higher similarity to that of *Halomonas* is also present in the genome (Table 4). This second BCCT gene was not differentially expressed under changing light regimes. *D. shibae* harbors genes for three different DMSP degradation pathways in its genome (Table 4). Laboratory experiments with [^2^H_6_]DMSP and derivatives had demonstrated that this organism employs both, the lytic pathway releasing DMS and the demethylation pathway releasing methylsulfide (MeSH) (Dickschat et al., 2010). However, none of these genes displayed a significant change in expression in our dataset when light and dark samples were compared. We therefore asked if the RNAseq-data could provide any hints which pathway is preferentially used by *D. shibae*. We found that the BCCT encoding gene as well as most genes with a role in DMSP catabolism had a very low coverage of mapped reads (Supplementary Material). The exceptions are *dddC*, encoding the malonate semialdehyde dehydrogenase of the lytic pathway and *dmdC*, encoding the 3-methylmercaptopropionyl-CoA dehydrogenase of the demethylation pathway, suggesting that both pathways may be simultaneously active.

**Table 4:** **Similarity of *D. shibae* genes involved in DMSP catabolism to reference organisms**.

**Reference oragnism**	**Gene symbol**	**GenBank**	**Description**	**Locus tag**	***E-value D. shibae***	**Identity (%)**
*Halomonas* sp. HTNK1	*dddT*	ACV84066.1	BCCT family DMSP transporter	Dshi_2119	2.0E−70	32
	*dddT*	ACV84066.1	BCCT family DMSP transporter	Dshi_0579	1.0E−55	33
	*dddD*	ACV84065.1	Putative DMSP CoA transferase	Dshi_3632	0.0E+00	43
	*dddA*	ACV84069.1	Putative 3-hydroxypropionate dehydrogenase	Dshi_0804	1.0E−148	45
	*dddC*	ACV84070.1	Putative malonate semialdehyde dehydrogenase	Dshi_1747	7.0E−179	53
*Sulfitobacter* sp. EE-36	*dddL*	ADK55772.1	DMSP lyase	Dshi_3313	9.0E−71	50
*Ruegeria pomeroyi* DSS-3	*dmdA*	YP_167148.1	DMSP demethylase	Dshi_2320	6.0E−149	63
	*dmdB*	YP_167275.1	3-methylmercaptopropionyl-CoA ligase	Dshi_0833	5.0E−70	33
	*dmdC*	YP_168992.1	3-methylmercaptopropionyl-CoA dehydrogenase	Dshi_0839	2.0E−156	43

It remains to be clarified which carbon sources are provided by *P. minimum* to *D. shibae* at this stage of the co-culture. Genes of the glycolate metabolism were not differentially expressed in the light. DMSP however seems a likely candidate. It is produced by *P. minimum* in considerable amounts (Caruana and Malin, 2014). The BCCT-family of proteins has been demonstrated to transport DMSP (Todd et al., 2010) and the encoding gene is not regulated by light when *D. shibae* is cultured alone (Tomasch et al., 2011). Therefore, the differential expression suggests a specific interaction with the algae, in particular the uptake of DMSP released by *P. minimum* in the light. As it has been shown for other microalgae, DMSP release is low during the exponential growth phase (Li et al., 2010), which might explain the slow growth of the bacteria during this stage. A few dying *P. minimum* cells might possibly also account for extracellular DMSP in the co-culture. It should also be considered that the BCCT proteins might be involved in the uptake of other released osmolytes. *P. minimum* produces in addition to DMSP the structurally related osmolytes glycine-betaine (GBT) and dimethylsulfonioacetate (DMS-Ac) to a considerable amount at the salt-concentration of 3.5% used in our study (Gebser and Pohnert, 2013).

## Conclusions

The co-culture of *D. shibae* and *P. minimum* can be divided into two phases, a mutualistic phase where both partner profit from each other and a pathogenic phase where *D. shibae* probably kills starved dinoflagellate cells. Our experiments further support the hypothesis of a “Jekyll and Hyde” lifestyle of *Roseobacters* that has first been proposed for *P. inhibens*.

It turned out that we were not able to substantially enrich bacterial mRNA in the early stage of the co-culture probably because of the low amount of bacterial cells and the large amount of algal ribosomal RNA. However, the obtained results were reproducible and allowed to draw first conclusions based on the most highly expressed or regulated genes.

We gained first insights into the transcriptome of viable but non-growing *D. shibae* cells in co-culture. We conclude that *D. shibae* actively maintains its PHA pool during the first days of cultivation. The extremely high expression of the phasin genes and the scarcity of external carbon sources might point to a degradation of the PHA bodies in the cell. The higher expression level of the *phaP* genes in the dark suggests that light serves as additional energy source during the day and therefore reduces the need to use PHAs as energy source. As the expression of photosynthesis genes was actively maintained in response to light we suggest that aerobic anoxygenic photosynthesis was functional. Thus, *D. shibae* might survive mainly on its stored carbon sources and light harvesting during this early phase of co-cultivation. The up-regulation of one BCCT gene in the light exclusively in the co-culture points to the uptake of osmolytes, in particular DMSP, released by *P. minimum*, although this might not be sufficient to provide *D. shibae* with enough carbon for growth. These hypotheses derived from the transcriptome data have to be further tested on the physiological level, e.g., by quantifying the PHA content in the cells and measuring the production of DMSP and other osmolytes in co-culture.

### Conflict of interest statement

The authors declare that the research was conducted in the absence of any commercial or financial relationships that could be construed as a potential conflict of interest.
