# Supplementary material for: A dual-species co-cultivation system to study the interactions between Roseobacters and dinoflagellates
Source: Front Microbiol. 2014 Jun 25;5:311. doi: 10.3389/fmicb.2014.00311 (PMC4069834; doi:10.3389/fmicb.2014.00311)
Supplement: Supplementary file 1 [file DataSheet1.DOCX]

Data Sheet 1. Mapped reads and expression values of the genes involved in PHA and DMSP methabolism.

|  | Locus tag | Gene symbol | Gene length | **replicate 1** | |  | **replicate 2** | |
| --- | --- | --- | --- | --- | --- | --- | --- | --- |
|  |  |  |  | Unique reads | RPKM |  | Unique reads | RPKM |
| **PHA metabolism** | Dshi_2230 | *phaR* | 565 | 40 | 168.3 |  | 10 | 68.2 |
|  | Dshi_2231 | *phaP_2_* | 433 | 294 | 1614.4 |  | 166 | 1476.7 |
|  | Dshi_2232 | *phaP_1_* | 451 | 4108 | 21657.0 |  | 2511 | 21445.2 |
|  | Dshi_2233 | *phaC* | 1819 | 73 | 95.4 |  | 46 | 97.4 |
|  | Dshi_2234 | *phaZ* | 1282 | 48 | 89.0 |  | 32 | 96.1 |
|  | Dshi_3066 | *phaA* | 1180 | 46 | 92.7 |  | 36 | 117.5 |
|  | Dshi_3067 | *phaB* | 727 | 34 | 111.2 |  | 41 | 217.2 |
| **DMSP metabolism** | Dshi_0579 | *dddT* | 1564 | 1 | 1.5 |  | 1 | 2.5 |
|  | Dshi_3632 | *dddD* | 2485 | 13 | 12.4 |  | 3 | 4.7 |
|  | Dshi_0804 | *dddA* | 1627 | 0 | 0 |  | 1 | 2.4 |
|  | Dshi_1747 | *dddC* | 1504 | 147 | 232.4 |  | 50 | 128.1 |
|  | Dshi_3312 | *dddL* | 682 | 10 | 34.9 |  | 8 | 45.2 |
|  | Dshi_2320 | *dmdA* | 1111 | 6 | 12.8 |  | 0 | 0 |
|  | Dshi_0833 | *dmdB* | 1630 | 2 | 2.9 |  | 4 | 9.5 |
|  | Dshi_0839 | *dmdC* | 1783 | 93 | 124.0 |  | 26 | 56.2 |
